# Supplementary material for: Childhood Diarrhea Prevalence and Uptake of Oral Rehydration Solution and Zinc Treatment in Nigeria
Source: Children (Basel). 2022 Nov 9;9(11):1722. doi: 10.3390/children9111722 (PMC9688883; doi:10.3390/children9111722)
Supplement: Supplementary file 1 [file children-09-01722-s001.zip › children-1941276-Supplementary.pdf]

## Supplementary

**Supplemental Table S1:** Period prevalence of diarrhea during the 2 weeks prior to the survey among children under 5 years of age in the 2018 NDHS (N=30,713)

| Characteristics | n | Percentage of under 5years old who had diarrhea reported during the 2 weeks before the survey (95% CI) | P-value |
|---|---|---|---|
| **Child's age in Months** | | | |
| < 6 | 3250 | 9.6 (8.6, 10.9) | |
| 6-11 | 3149 | 20.1 (18.5, 21.7) | |
| 12-23 | 6059 | 20.2 (19.1, 21.4) | |
| 24-35 | 5834 | 13.8 (12.8, 14.9) | <0.001 |
| 36-47 | 6168 | 8.7 (7.9, 9.5) | |
| 48-59 | 6253 | 6.1 (5.9, 7.4) | |
| **Sex of child** | | | |
| Male | 15537 | 12.7 (12.1, 13.3) | |
| Female | 15176 | 12.9 (12.3, 13.5) | 0.766 |
| **Birth order** | | | |
| First | 5885 | 11.6 (10.7, 12.6) | |
| Second/Third | 10504 | 11.5 (10.8, 12.3) | <0.001 |
| Fourth/Fifth | 7202 | 12.2 (11.3, 13.1) | |
| Sixth and above | 7122 | 16.3 (15.3, 17.3) | |
| **Child lives with whom** | | | |
| Respondent | 29895 | 13.0 (12.6, 13.5) | <0.001 |
| Lives elsewhere | 818 | 3.6 (2.5, 5.4) | |
| **Place of Delivery** | | | |
| Non-institutional | 18277 | 15.1 (14.5, 15.7) | <0.001 |
| Institutional | 12436 | 9.3 (8.8, 9.9) | |
| **Maternal age (in years)** | | | |
| < 20 | 1289 | 20.1 (17.6, 22.8) | |
| 20-34 | 21583 | 12.8 (12.3, 13.3) | <0.001 |
| 35-49 | 7844 | 11.6 (10.8, 12.4) | |
| **Current marital status** | | | |
| Never in union | 645 | 10.9 (8.0, 14.6) | |
| Married/living with partner | 29181 | 12.8 (12.4, 13.3) | 0.585 |
| Widowed/Divorced/Separated | 887 | 12.4 (10.0, 15.5) | |
| **Maternal educational level** | | | |
| No education | 13527 | 16.3 (15.6,17.0) | |
| Primary | 4776 | 13.0 (12.0, 14.2) | |
| Secondary | 9913 | 9.5 (8.8, 10.2) | |
| Higher | 2497 | 6.3 (5.3, 7.5) | <0.001 |
| **Mother currently working** | | | |
| No | 9949 | 13.3 (12.5, 14.0) | 0.250 |
| Yes | 20764 | 12.6 (12.1, 13.1) | |
| **Place of residence** | | | |
| Urban | 10851 | 9.6 (9.0,10.2) | <0.001 |
| Rural | 19862 | 14.9 (14.3, 15.5) | |
| **Religion of mother** | | | |
| Catholic | 2744 | 7.4 (6.5, 8.5) | |
| Other Christian | 9594 | 7.3 (6.7, 7.9) | |
| Islam | 18113 | 16.1 (15.5, 16.8) | <0.001 |
| Traditionalist | 108 | 5.8 (2.9,11.4) | |
| Other | 154 | 1.8 (0.4,7.0) | |
| **Ethnicity of mother** | | | |
| Hausa | 9576 | 15.8 (15.0, 16.6) | |
| Fulani | 2884 | 22.4 (20.8, 24.2) | |
| Ekoi | 144 | 6.9 (3.5, 13.2) | |
| Ibibio | 440 | 7.9 (5.4, 11.4) | |
| Igala | 288 | 7.1(4.0, 12.6) | |
| Igbo | 4200 | 6.1 (5.3, 6.9) | |
| Ijaw/Izion | 750 | 3.6 (2.2, 5.9) | |
| Kanuri/Beriberi | 746 | 13.2 (10.8, 16.1) | |
| Tiv | 723 | 9.8 (7.6, 12.4) | |
| Yoruba | 3037 | 5.7 (4.9, 6.7) | <0.001 |
| Other | 7897 | 13.4 (12.6, 14.3) | |
| Don't know | 28 | 4.9 (0.7, 27.6) | |
| **Sources of drinking water** | | | |
| Unimproved | 12058 | 15.1 (14.4, 15.8) | <0.001 |
| Improved | 18655 | 11.5 (11.0, 12.0) | |

| | | | | |
|---|---|---|---|---|
| **Toilet facility** | | | | |
| | Unimproved | 15644 | 14.3 (13.6, 14.9) | <0.001 |
| | Improved | 15069 | 11.4 (10.8, 12.0) | |
| **Presence of water at hand washing place (n=24304)** | | | | |
| | Water not available | 9577 | 14.8 (14.0, 15.7) | <0.001 |
| | Water available | 14727 | 11.2 (10.7, 11.8) | |
| **Soap or detergent present at hand washing place (n=24304)** | | | | |
| | No | 16658 | 14.5 (13.9, 15.2) | <0.001 |
| | Yes | 7646 | 9.0 (8.3, 9.8) | |
| **Sex of household head** | | | | |
| | Male | 27776 | 13.1 (12.6, 13.5) | 0.001 |
| | Female | 2937 | 10.0 (8.8, 11.4) | |
| **Household wealth quintiles** | | | | |
| | Poorest | 7081 | 18.6 (17.6. 19.7) | <0.001 |
| | Poorer | 6839 | 15.4 (14.5, 16.4) | |
| | Middle | 6509 | 12.2 (11.3, 13.1) | |
| | Richer | 5747 | 9.7 (8.8, 10.7) | |
| | Richest | 4537 | 6.2 (5.4, 7.0) | |
| **Region** | | | | |
| | North Central | 5403 | 11.4(10.4,12.4) | <0.001 |
| | North East | 6481 | 24.6 (23.4, 25.9) | |
| | North West | 8934 | 13.8 (13.0, 14.6) | |
| | South East | 3545 | 6.1(5.3.7.0) | |
| | South South | 3021 | 6.1 (5.1, 7.2) | |
| | South West | 3329 | 5.2 (4.4, 6.1) | |
| **Diarrrhea status 2 weeks before Survey** | | | | |
| | Did not have diarrhea | 26757 | 87.1 (86.7, 87.4) | - |
| | Had diarrhea | 3965 | 12.9 (12.5, 13.3) | |

**Supplemental Table S2:** Proportion of children under 5 years of age with diarrhea for whom mothers/caregivers reported the child received ORS, zinc and ORS and zinc in the 2018 NDHS (N=3956)

| Characteristics | | n | Percentage of ORS uptake among under 5 years who had diarrhea 2 weeks before the survey (95% CI) | Percentage of zinc supplement uptake among under 5 years who had diarrhea 2 weeks before the survey (95% CI) | Percent of ORS and zinc supplement uptake among under 5 years who had diarrhea 2 weeks before the survey (95% CI) |
|---|---|---|---|---|---|
| **Child's age in Months** | | | | | |
| | < 6 | 324 | 33.0 (27.4, 39.2) | 20.3 (15.6, 26.1) | 15.4 (11.2, 20.9) |
| | 6-11 | 641 | 40.3 (36.0, 44.7) | 32.0 (27.8, 36.4) | 22.8 (19.3, 26.8) |
| | 12-23 | 1229 | 46.8 (43.6, 50.0) | 33.7 (30.8, 36.8) | 25.9 (23.2, 28.8) |
| | 24-35 | 800 | 42.7 (38.8, 46.7) | 32.1 (28.5, 35.9) | 24.5 (21.2, 28.1) |
| | 36-47 | 560 | 39.4 (34.9, 44.1) | 29.4 (25.3, 33.8) | 21.8 (18.2, 25.9) |
| | 48-59 | 402 | 38.2 (32.8, 43.8) | 30.6 (25.4, 36.2) | 23.3 (18.9, 28.4) |
| **Sex of child** | | | | | |
| Male | | 2015 | 41.5 (39.1, 44.0) | 30.3 (28.0, 32.6) | 21.6 (19.6, 23.8) |
| Female | | 1941 | 42.2 (39.8, 44.8) | 32.0 (29.6, 34.5) | 25.4 (23.2, 27.6) |
| **Birth order** | | | | | |
| First | | 684 | 39.6 (35.4, 43.9) | 27.7 (23.8, 31.9) | 20.2 (16.9, 23.9) |
| Second/Third | | 1228 | 43.8 (40.6, 47.1) | 31.6 (28.6, 34.7) | 23.7 (21.0, 26.6) |
| Fourth/Fifth | | 883 | 40.7 (37.1, 44.4) | 33.0 (29.5, 36.6) | 24.8 (21.7, 28.2) |
| Sixth and above | | 1161 | 42.1 (38.9, 45.4) | 31.3 (28.4, 34.4) | 24.2 (21.5, 27.1) |
| **Place of Delivery** | | | | | |
| Non-institutional | | 2776 | 37.5 (35.5, 39.6) | 28.6 (26.7, 30.6) | 21.0 (19.4, 22.8) |
| Institutional | | 1180 | 52.4 (49.1, 55.7) | 37.1 (33.9, 40.4) | 29.3 (26.3, 32.6) |
| **Maternal age (in years)** | | | | | |
| | < 20 | 246 | 32.5 (26.3, 39.5) | 25.8 (19.4, 33.4) | 16.3 (11.7, 22.3) |
| | 20-34 | 2805 | 43.7 (41.6, 45.9) | 31.7 (29.8, 33.8) | 24.3 (22.5, 26.2) |

| | | | | |
|---|---|---|---|---|
| 35-49 | 905 | 38.8 (35.3, 42.5) | 30.8 (27.5, 34.3) | 23.0 (20.0, 26.2) |
| **Current marital status** | | | | |
| Never in union | 59 | 41.4 (26.4, 58.2) | 17.6 (9.0, 31.6) | 12.2(5.1, 26.2) |
| Married/living with partner | 3799 | 41.8 (40.0, 43.6) | 31.4 (29.7, 33.1) | 23.7 (22.2, 25.3) |
| Widowed/Divorced/Separated | 98 | 44.3 (32.8, 56.4) | 29.9 (20.0, 42.1) | 20.8 (12.8, 32.0) |
| **Maternal educational level** | | | | |
| No education | 2248 | 38.9 (36.6, 41.2) | 28.7 (26.6, 30.9) | 22.1 (20.2, 24.2) |
| Primary | 614 | 44.9 (40.4, 49.4) | 28.9 (25.1, 33.1) | 22.4 (18.9, 26.3) |
| Secondary | 931 | 44.3 (40.6, 48.1) | 35.8 (32.1, 39.6) | 24.8 (21.7, 28.3) |
| Higher | 163 | 58.6 (49.9, 66.8) | 45.6 (37.1, 54.3) | 37.6 (29.5, 46.3) |
| **Mother currently working** | | | | |
| No | 1353 | 36.0 (33.1, 39.0) | 28.7 (26.0, 31.6) | 20.0 (17.6, 22.6) |
| Yes | 2603 | 44.8 (42.6, 47.0) | 32.3 (30.3, 34.4) | 25.2 (23.4, 27.2) |
| **Place of residence** | | | | |
| Urban | 1098 | 51.6(48.2, 55.0) | 37.8 (34.5, 41.1) | 30.2 (27.2, 33.4) |
| Rural | 2858 | 37.8 (35.8. 39.9) | 28.3 (26.4, 30.3) | 20.6 (19.0, 22.4) |
| **Religion of mother** | | | | |
| Catholic | 215 | 43.5 (36.5, 50.6) | 26.4 (20.7, 33.1) | 19.5 (14.4, 25.6) |
| Other Christian | 722 | 41.6 (37.5, 45.9) | 28.3 (24.6, 32.4) | 18.9 (15.7, 22.5) |
| Islam | 3009 | 41.9 (39.9, 43.9) | 32.0 (30.1, 34.0) | 24.7 (22.9, 26.5) |
| Traditionalist | 8 | 30.7 (8.2, 68.7) | 15.7 (02, 60.4) | 15.7 (2.2, 60.4) |
| Other | 2 | 45.1 (4.9, 92.9) | - | 45.1 (4.9, 92.9) |
| **Ethnicity of mother** | | | | |
| Hausa | 1511 | 44.6 (41.8, 47.4) | 37.8 (35.1, 40.5) | 28.6 (26.2, 31.2) |
| Fulani | 675 | 29.8(25.9, 34.0) | 20.9 (17.6, 24.5) | 14.5 (11.8, 17.8) |
| Ekoi | 10 | 66.6 (34.2, 88.4) | 52.4 (21.5, 81.6) | 42.8 (16.5, 74.0) |
| Ibibio | 32 | 37.2 (20.1, 58.3) | 34.0 (17.4, 55.7) | 26.8 (11.6, 50.5) |
| Igala | 17 | 55.7 (27.4, 80.7) | 38.5 (14.2, 70.4) | 33.8 (10.9, 68.1) |
| Igbo | 276 | 50.4 (43.9, 57.0) | 29.8 (24.0, 36.2) | 23.7 (18.6, 29.8) |
| Ijaw/Izion | 19 | 54.0 (30.0, 76.3) | 63.6 (37.6, 83.5) | 45.1 (23.4, 68.7) |
| Kanuri/Beriberi | 113 | 55.8 (45.6, 65.5) | 28.7 (19.5, 40.1) | 26.1 (17.1, 37.7) |
| Tiv | 68 | 47.4 (34.9, 60.2) | 25.1 (15.6, 37.8) | 16.5 (9.0, 28.4) |
| Yoruba | 186 | 47.8 (39.6, 56.1) | 30.3 (22.9, 38.8) | 23.6 (17.1, 31.7) |
| Other | 1048 | 38.0 (34.6, 41.6) | 25.0 (22.0, 28.2) | 18.3 (157, 21.1) |
| **Sources of drinking water** | | | | |
| Unimproved | 1814 | 33.9 (31.4, 36.5) | 25.2 (22.9, 27.7) | 17.9 (16.0, 20.1) |
| Improved | 2142 | 44.7 (45.3, 50.2) | 35.4 (33.1, 37.8) | 27.5 (25.4, 29.7) |
| **Sex of household head** | | | | |
| Male | 3685 | 41.8 40.0, 43.6) | 31.6 (29.9, 33.4) | 23.8 (22.2, 25.4) |
| Female | 271 | 43.1 (36.3, 50.2) | 24.6 (19.1, 31.0) | 19.4 (14.5, 25.5) |
| **Household wealth quintiles** | | | | |
| Poorest | 1303 | 35.2 (32.2, 38.2) | 25.9 (23.2, 28.7) | 19.7 (17.3, 22.3) |
| Poorer | 1031 | 38.0 (34.6, 41.4) | 25.2 (22.2, 28.4) | 19.3 (16.7, 22.3) |
| Middle | 776 | 43.1 (39.2, 47.1) | 36.1 (32.2, 40.1) | 25.5 (22.2, 29.1) |
| Richer | 552 | 48.6 (43.8, 53.5) | 38.7 (34.1, 43.6) | 28.5 (24.4, 33.0) |
| Richest | 294 | 65.3 (59.0, 71.2) | 45.2 (38.8, 51.7) | 37.9 (31.4, 44.0) |

| Region | N | | | |
|---|---|---|---|---|
| North Central | 540 | 40.8(36.2, 45.5) | 22.9 (19.3, 27.1) | 17.9 (14.6, 21.8) |
| North East | 554 | 38.2 (35.3, 41.1) | 22.0 (19.5, 24.6) | 19,9 (15.6, 20.4) |
| North West | 1580 | 43.4(40.4,46.4) | 41.9 (38.9, 44.9) | 30.0 (27.3, 32.8) |
| South East | 1234 | 44.9 (38.2, 51.8) | 27.5, (21.8, 34.1) | 22.9 (17.6, 29.3) |
| South South | 232 | 44.6 (36.3, 53.1) | 32.7 (25.4, 41.1) | 22.6 (16.5, 30.1) |
| South West | 162 | 52.9 (44.7, 60.9) | 35.1 (27.5, 43.6) | 27.5 (20.6, 35.8) |

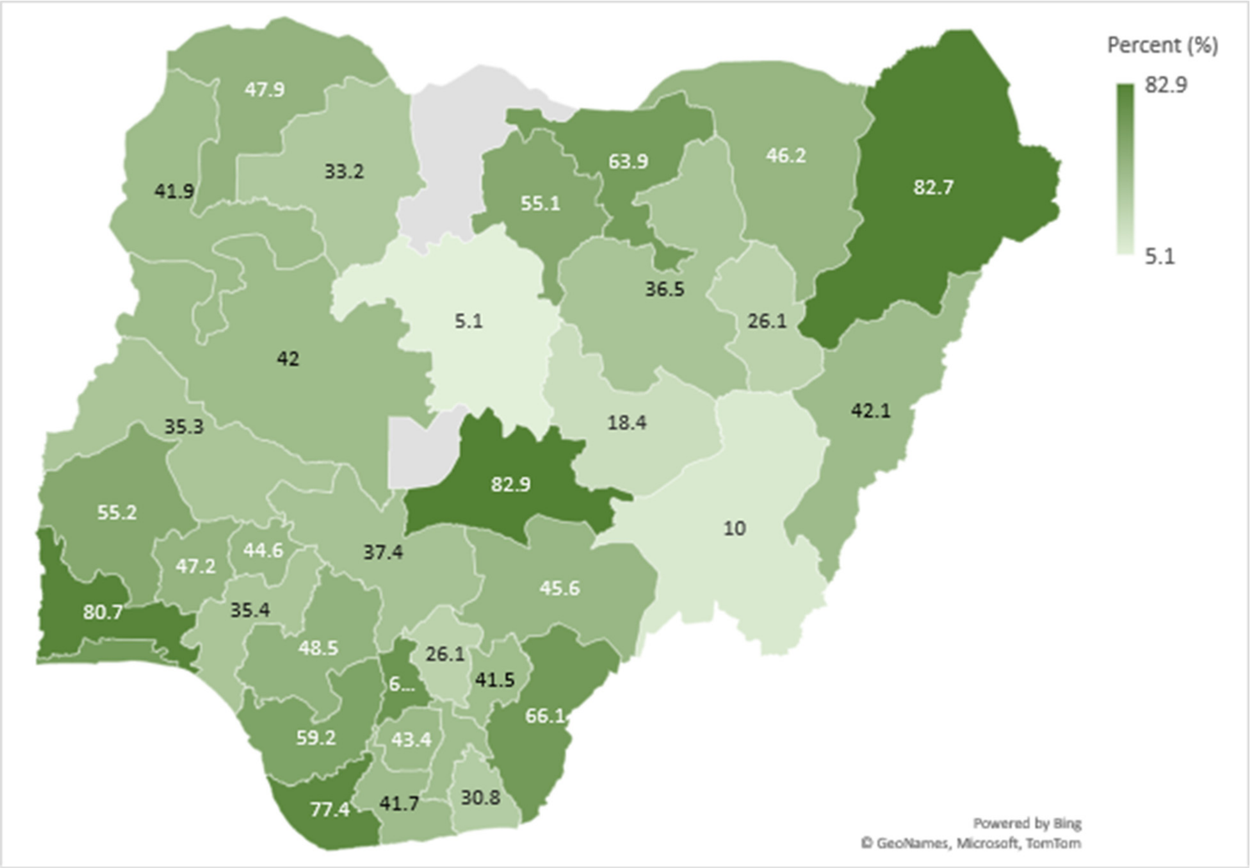


**Supplemental Figure S1:** Percentage of children with diarrhea during the 2 weeks prior to the survey that were reported to have received oral rehydration solution (ORS) by state in the 2018 NDHS

**Supplemental Table S3:** Univariable regression models to estimate the likelihood of uptake of Zinc and ORS by under 5 children, maternal and household characteristics during episode of childhood diarrhea in the NDHS 2018

| Characteristics | N | Zinc | | ORS | | Zinc & ORS | |
|---|---|---|---|---|---|---|---|
| | | Univariable relative risk 95% CI | p-value | Univariable relative risk 95% CI | p-value | Univariable relative risk 95% CI | p-value |
| **Child's age in Months** | | | | | | | |
| **< 6** | 324 | Reference | | Reference | | Reference | |
| **6-11** | 641 | 1.66 (1.23. 2.23) | 0.001 | 1.38 (1.09, 1.75) | 0.007 | 1.62 (1.15, 2.28) | 0.006 |
| **12-23** | 1229 | 1.82(1.38, 2.41) | <0.001 | 1.52 (1.22,1.90) | <0.001 | 1.83 (1.33, 2.52) | <0.001 |
| **24-35** | 800 | 1.71 (1.28, 2.28) | 0.0061 | 1.38 (1.10, 1.74) | 0.006 | 1.69 (1.21, 2.35) | 0.002 |
| **36-47** | 560 | 1.60 (1.18, 2.17) | 0.002 | 1.27 (1.00, 1.62) | 0.051 | 1.55 (1.09, 2.20) | 0.015 |
| | 402 | 1.63 (1.18, 2.23) | 0.003 | 1.29 (1.00, 1.66) | 0.056 | 1.65 (1.15, 2.38) | 0.007 |

| | | | | | | | |
|---|---|---|---|---|---|---|---|
| **48-59** | | | | | | | |
| **Sex of child** | | | | | | | |
| Male | 2015 | Reference | | Reference | | Reference | |
| Female | 1941 | 1.00 (0.89, 1.13) | 0.941 | 1.0 (0.91, 1.10) | 0.988 | 1.11 (0.97, 1.27) | 0.116 |
| **Birth order** | | | | | | | |
| First | 684 | Reference | | Reference | | Reference | |
| Second/Third | 1228 | 1.09 (0.91, 1.31) | 0.321 | 1.10(0.95, 1.28) | 0.199 | 1.13 (0.92, 1.49) | 0.250 |
| Fourth/Fifth | 883 | 1.21 (1.00, 1.46) | 0.049 | 1.05 (0.90, 1.24) | 0.515 | 1.26 (1.02, 1.57) | 0.036 |
| Sixth and above | 1161 | 1.12 (0.94, 1.34) | 0.211 | 1.05 (0.90, 1.22) | 0.570 | 1.16 (0.94, 1.43) | 0.168 |
| **Place of Delivery** | | | | | | | |
| Non-institutional | 2776 | Reference | | Reference | | Reference | |
| Institutional | 1180 | 1.28 (1.14, 1.45) | <0.001 | 1.35 (1.22, 1.50) | <0.001 | 1.27 (1.111.47) | 0.001 |
| **Maternal age (in years)** | | | | | | | |
| < 20 | 246 | Reference | | Reference | | Reference | |
| 20-34 | 2805 | 1.36 (1.03, 1.79) | 0.029 | 1.26 (1.01, 1.59) | 0.043 | 1.54 (1.10, 2.16) | 0.011 |
| 35-49 | 905 | 1.31 (0.98, 1.76) | 0.068 | 1.15 (0.90, 1.47) | 0.255 | 1.46 (1.02, 2.08) | 0.038 |
| **Current marital status** | | | | | | | |
| Never in union | 59 | Reference | | Reference | | Reference | |
| Married/living with partner | 3799 | 1.57 (0.87, 2.85) | 0.136 | 1.24 (0.79, 1.95) | 0.353 | 1.85 (0.88, 3.90) | 0.104 |
| Widowed/Divorced/Separated | 98 | 1.48 (0.73, 2.98) | 0.275 | 1.17 (0.67, 2.04) | 0.573 | 1.63 (0.69, 3.89) | 0.267 |
| **Maternal educational level** | | | | | | | |
| No education | 2248 | Reference | | Reference | | Reference | |
| Primary | 614 | 1.00 (0.84, 1.18) | 0.967 | 1.11 (0.96, 1.28) | 0.146 | (0.82, 1.21) | 0.948 |
| Secondary | 931 | 1.21 (1.06, 1,.39) | 0.005 | 1.16 (1.03, 1.30) | 0.017 | 1.09 (0.93, 1.29) | 0.279 |
| Higher | 163 | 1.54 (1.20, 1.98) | 0.001 | 1.51 (1.22, 1.86) | <0.001 | 1.62 (1.22, 2.14) | 0.001 |
| **Mother currently working** | | | | | | | |
| No | 1353 | Reference | | Reference | | Reference | |
| Yes | 2603 | 1.12 (0.99, 1.27) | 0.066 | 1.21 (1.09,1.35) | <0.001 | 1.29 (1.11. 1.49) | 0.001 |
| **Place of residence** | | | | | | | |
| Urban | 1098 | Reference | | Reference | | Reference | |
| Rural | 2858 | 0.72 (0.64, 0.82) | <0.001 | 0.75 (0.68, 0.84) | <0.001 | 0.69 (0.60, 0.80) | <0.001 |
| **Religion** | | | | | | | |
| Catholic | 215 | Reference | | Reference | | Reference | |
| Other Christian | 722 | 0.99 (0.74, 1.34) | 0.971 | 0.93 (0.74, 1.18) | 0.562 | 0.87 (0.61, 1.24) | 0.444 |
| Islam | 3009 | 1.15 (0.88, 1.51) | 0.296 | 0.94 (0.76, 1.16) | 0.548 | 1.18 (0.87, 1.61) | 0.292 |
| Traditionalist | 8 | 0.48 (0.07, 3.47) | 0.467 | 0.59 (0.15, 2.40) | 0.461 | 0.64 (0.09, 4.65) | 0.659 |
| Other | 2 | 3.84 (0.94,15.73) | 0.062 | 1.18 (0.16, 8.48) | 0.868 | 2.56 (0.35, 18.59) | 0.353 |

| | | | | | | | |
|---|---|---|---|---|---|---|---|
| **Ethnicity** | | | | | | | |
| Hausa | 1511 | Reference | | Reference | | Reference | |
| Fulani | 675 | 0.54 (0.45, 0.65) | <0.001 | 0.60 (0.51, 0.71) | <0.001 | 0.47 (0.37, 0.59) | <0.001 |
| Ekoi | 10 | 1.60 (0.72, 3.59) | 0.249 | 1.33 (0.60, 2.98) | 0.481 | 1.74 (0.72. 4.20) | 0.218 |
| Ibibio | 32 | 0.67 (0.33, 1.34) | 0.258 | 0.63 (0.32, 1.21) | 0.163 | 0.54 (0.22, 1.31) | 0.176 |
| Igala | 17 | 0.79 (0.33, 1.90) | 0.593 | 1.18 (0.61, 2.27) | 0.625 | 0.82 (0.31, 2.19) | 0.691 |
| Igbo | 276 | 0.75 (0.59, 0.95) | 0.016 | 1.08 (0.90,1.30) | 0.413 | 0.78 (0.60, 1.02) | 0.070 |
| Ijaw/Izon | 19 | 1.83 (1.06, 3.17) | 0.031 | 1.29 (0.71, 2.34) | 0.405 | 1.83 (0.98, 3.43) | 0.058 |
| Kanuri/beriberi | 113 | 0.57 (0.38, 0.85) | <0.007 | 1.02 (0.77, 1.36) | 0.869 | 0.62 (0.39, 0.96) | 0.034 |
| Tiv | 68 | 0.59 (0.35, 0.99) | 0.044 | 0.92 (0.63, 1.34) | 0.650 | 0.51 (0.27, 0.96) | 0.036 |
| Yoruba | 186 | 0.69 (0.51, 0.93) | 0.014 | 0.99 (0.79, 1.25) | 0.952 | 0.69 (0.50. 0.97) | 0.032 |
| Other | 1048 | 0.65 (0.56, 0.75) | <0.001 | 0.80 (0.71, 0.91) | 0.001 | 0.61 (0.51, 0.73) | <0.001 |
| **Sources of drinking water** | | | | | | | |
| Unimproved | 1814 | Reference | | Reference | | Reference | |
| Improved | 2142 | 1.50 (1.34, 1.70) | <0.001 | 1.51 (1.36, 1.67) | <0.001 | 1.63 (1.42, 1.88) | <0.001 |
| **Sex of household head** | | | | | | | |
| Male | 3685 | Reference | | Reference | | Reference | |
| Female | 271 | 0.84 (0.66, 1.08) | 0.167 | 0.96 (0.79, 1.18) | 0.713 | 0.84 (0.63, 1.11) | 0.223 |
| **Wealth quintiles** | | | | | | | |
| Poorest | 1303 | Reference | | Reference | | Reference | |
| Poorer | 1031 | 0.99 (0.83, 1.17) | 0.880 | 1.10 (0.95, 1.26) | 0.194 | 0.97 (0.80, 1.17) | 0.734 |
| Middle | 776 | 1.43 (1.21, 1.68) | <0.001 | 1.32 (1.14, 1.52) | <0.001 | 1.36 (1.13, 1.65) | 0.002 |
| Richer | 552 | 1.66 (1.39, 1.97) | <0.001 | 1.45 (1.24, 1.69) | <0.001 | 1.56 (1.28, 1.92) | <0.001 |
| Richest | 294 | 1.90 (1.55, 2.33) | <0.001 | 1.95 (1.65, 2.23) | <0.001 | 1.97 (1.56, 2.48) | <0.001 |
| **Region** | | | | | | | |
| North Central | 540 | Reference | | Reference | | Reference | |
| North East | 554 | 0.85 (0.69, 1.05) | 0.127 | 0.82 (0.70, 0.96) | 0.016 | 0.85 (0.67, 1.08) | 0.181 |
| North West | 1580 | 1.87 (1.54, 2.27) | <0.001 | 1.16 (0.99,1.36) | 0.062 | 1.79 (1.43, 2.23) | <0.001 |
| South East | 1234 | 1.16 (0.85, 1.57) | 0.347 | 1.13 (0.90, 1.43) | 0.291 | 1.22 (0.87, 1.71) | 0.256 |
| South South | 232 | 1.55 (1.14, 2.11) | 0.006 | 1.14 (0.87, 1.49) | 0.333 | 1.47 (1.02, 2.10) | 0.038 |
| South West | 162 | 1.29 (0.95, 1.76) | 0.103 | 1.25 (0.98, 1.59) | 0.067 | 1.28 (0.90, 1.83) | 0.171 |
